# Formyl Peptide Receptor Activation Elicits Endothelial Cell Contraction and Vascular Leakage

**DOI:** 10.3389/fimmu.2016.00297

**Published:** 2016-08-02

**Authors:** Camilla F. Wenceslau, Cameron G. McCarthy, R. Clinton Webb

**Affiliations:** ^1^Department of Physiology, Augusta University, Augusta, GA, USA

**Keywords:** formyl peptide receptor, mitochondria *N*-formyl peptides, endothelial cells, vascular leakage, SIRS

## Abstract

The major pathophysiological characteristic of systemic inflammatory response syndrome (SIRS) and sepsis is the loss of control of vascular tone and endothelial barrier dysfunction. These changes are attributed to pro-inflammatory mediators. It has been proposed that in patients and rats without infection, cell components from damaged tissue are the primary instigators of vascular damage. Mitochondria share several characteristics with bacteria, and when fragments of mitochondria are released into the circulation after injury, they are recognized by the innate immune system. *N*-Formyl peptides are common molecular signatures of bacteria and mitochondria and are known to play a role in the initiation of inflammation by activating the formyl peptide receptor (FPR). We have demonstrated that infusion of mitochondrial *N*-formyl peptides (F-MIT) leads to sepsis-like symptoms, including vascular leakage. We have also observed that F-MIT, *via* FPR activation, elicits changes in cytoskeleton-regulating proteins in endothelial cells. Therefore, we hypothesize that these FPR-mediated changes in cytoskeleton can cause endothelial cell contraction and, consequently vascular leakage. Here, we propose that endothelial FPR is a key contributor to impaired barrier function in SIRS and sepsis patients following trauma.

## Introduction

Systemic inflammatory response syndrome (SIRS) and sepsis are the principal causes of death in intensive care units, with mortality rates between 30 and 70% (1–3) The diagnosis of sepsis requires confirmation of bacterial growth in blood cultures, as well as the presence of two or more of the following symptoms: hypothermia or hyperthermia, tachycardia, tachypnea, and leukocytopenia or leukocytosis (1, 2). It is noteworthy that only about one-third to one-half of patients meeting these criteria were subsequently confirmed to have sepsis (1–3). Accordingly, all remaining patients are diagnosed with SIRS, a sepsis-like condition. Therefore, diagnosis of SIRS requires the same symptoms, minus the presence of infection. One major pathophysiological characteristic of SIRS and sepsis is the breakdown of the endothelial barrier function results in the loss of fluid into the extravascular space that leads to edema in several tissues (1, 2, 4).

The circulatory responses to sepsis are characterized by an initial hyperdynamic phase, where there is a reduction in systemic vascular resistance due to peripheral vasodilation and unresponsiveness to vasoconstrictor drugs are observed (5). Later, the myocardial dysfunction caused by pro-inflammatory mediators leads to a hypodynamic phase, where cardiac output significantly falls and both the systolic and the diastolic functions of the heart are greatly impaired (5). The endothelium lining of the vascular walls is a major target of sepsis, especially during the initial hyperdynamic phase, and damage of endothelial cells accounts for much of the pathology of septic shock (4, 5). The increase in endothelial permeability and dysfunction has been associated with several factors, including hypoxia, oxidative stress and augmented TNF-α levels (4). Other interleukins (e.g., IL-1, IL-2, and IL-6) are also contributors (6). In line, IL-6 is known to be a clinically suitable biomarker for sepsis, and Waage and coworkers (7) observed high levels of IL-6 and its association with sepsis in patients with meningococcal infection (7). Corroborating these data, Lo and others (8) demonstrated that IL-6 induced redistribution of vascular-endothelial cadherin, which is the major component of endothelial adhesion and barrier function *via* adherens junctions. Vascular endothelial cadherin-regulated protein complexes that join adjacent endothelial cells and prevent leukocyte emigration and vascular leakage (6, 8). Therefore, disruption of vascular endothelial cadherins function results in trans-endothelial flow of fluid and interstitial edema. Also, it has been observed that other molecules released during acute inflammation such as bradykinin, thrombin, VEGF, and histamine result in endothelial activation and massive increases in glycocalyx expression of endothelial leukocyte adhesion molecule 1, intercellular adhesion molecule 1 (ICAM-1), and vascular cell adhesion molecule 1 (VCAM-1) (9). The increased expression of these proteins leads to leukocyte rolling, adherence, and migration, which initiate the inflammatory damage to endothelium and endo organs (9). Furthermore, it is well known that exacerbated production of nitric oxide by the inducible form of nitric oxide synthase (iNOS) contributes to vascular leakage and hyporeactivity. Nevertheless, pharmacological interventions using NOS inhibitors have not been successful. Currently, there are no therapies for blocking vascular leakage in SIRS and sepsis, given that the molecular mechanisms regulating vascular permeability are not completely understood.

### Contraction of Endothelial Cell: A New Paradigm for the Regulation of Vascular Leakage

Although the concept that active contraction of endothelial cells was first suggested by Majno in 1961 (10, 11), currently the intracellular events regulating endothelial contractile activity is still unknown. Just like in other type of cells, the dynamic assembly, disassembly, and reorganization of the actin and myosin cytoskeleton regulate endothelial cells contraction (12). Accordingly, Goeckeler and Wysolmerski (12) reported that thrombin stimulation results in rapid sustained isometric contraction in endothelial cells that increases twofold within 5 min and remains elevated for 60 min. Also, they observed that myosin light chain (MLC) phosphorylation precedes the development of isometric tension (12). Supporting these data, it has been shown that transfection of constitutively active MLCK induces MLC phosphorylation associated with increase in permeability in endothelial cells (13). On the other hand, inhibition of MLC phosphorylation with an MLCK antagonist greatly attenuates the increase in venular permeability in response to soluble inflammatory mediators (14).

More recently, it has been demonstrated that not only the interaction of actin and myosin is necessary for endothelial contraction but also changes in actin polymerization have been associated with the development of isometric force. In many cell types, including endothelial cells, the actin cytoskeleton is a highly dynamic structure that undergoes polymerization and depolymerization based upon cellular demand (15). It is known that actin polymerization occurs in two steps, nucleation and elongation. Nucleation occurs when three actin monomers bind together and provides a site for elongation. Elongation occurs when ATP bound globular (G)-actin binds and grows to form filamentous (F)-actin (15). Reorganization of F-actin, which is a fundamental unit for actin-based cytoskeleton structures, is paramount for endothelial cell contraction and barrier function. Several permeability factors, including angiogenic and inflammatory mediators, trigger signaling pathway in endothelial cell that enhance F-actin polymerization and actomyosin contractility.

It well known that Rho family of p21 small GTP-binding proteins are associated with the direct regulation of actin cytoskeleton (16). RhoA induces actin polymerization at focal adhesions by activating formin-homology protein Dia1, a potent activator of nucleation and elongation of actin filaments, and inhibits actin filament disassembly by inactivation of ADF/cofilin, a family of actin-binding proteins, which disassembles actin filaments. Additionally, RhoA promotes contractility by activating the myosin light-chain kinase through ROCK kinase (17). An interesting study by Gorovoy et al. (18) provide strong evidence that increased RhoA activity results in vascular leakage in mouse lung. In this study, it was observed that increased RhoA activity due to deletion of one its inhibitory proteins, RhoGDI, causes a loss of endothelial junctional integrity and breakdown in endothelial barrier function (18).

Mitogen-activated protein (MAP) kinases are a family of stress activated enzymes that initiate signaling cascades in response to several stimuli, including inflammation and injury. It has previously shown that p38 MAPK kinase leads to reorganization of the actin cytoskeleton to form stress fibers and increase in vascular permeability (19). Furthermore, It was demonstrated in a recently study by using atomic force microscopy and a combination of confocal microscopy methods that thermal injury induces venular hyperpermeability and that serum from burned rats induces endothelium cells actin rearrangement and contraction (20). However, inhibition of the p38 MAPK ameliorates resulting vascular dysfunction by significantly reducing endothelial cells contraction (20).

Despite new information about the pathophysiology of molecular mechanisms regulating vascular permeability, this disruption continues to be treated inappropriately and this contributes to an unacceptably high mortality rate in patients with SIRS and sepsis. Therefore, understanding vascular function and the causes for this disturbance would ultimately provide starting points for therapies designed to treat these devastating diseases.

### Mitochondrial *N*-Formyl Peptides, Formyl Peptide Receptor, and Endothelial Cell Cytoskeleton

It has been proposed that in patients and rats without infection, cell components from damaged tissue can initiate the genesis of SIRS (21–25). These cell components are collectively called damage-associated molecular patterns (DAMPs). DAMPs are endogenous molecules that are released from cells following injury. For evolutionary reasons, mitochondria share several characteristics with bacteria, and when fragments of mitochondria are released into the circulation, they are recognized by the innate immune system. Due to protein translation initiation by formyl-methionine in both bacteria and mitochondria, *N*-formyl peptides are common molecular signatures of bacteria and mitochondria and are known to play a role in the initiation of inflammation by activating the formyl peptide receptor (FPR) (22, 26, 27). The FPR has been identified as a subfamily of G-protein-coupled receptors (27). FPR-1 and FPR-2 are expressed at high levels on leukocytes and mediate cell chemotaxis (27). However, it has been demonstrated that mitochondrial DAMPs suppress pulmonary immune response *via* FPR-1 and -2 (28). FPR activation leads to cytoskeletal rearrangements, making *N*-formyl peptides potent inducers of neutrophil F-actin formation. Stimulation of human neutrophils with the formylated peptide (fMLP, bacteria derived), known to result in a prompt rise of the calcium, also induced a rapid decrease of G-actin content and increase of filamentous actin, F-actin, content (29). A reduction of the fMLP induced calcium transient to about 25 nM, resulted in a less pronounced decrease of G-actin content and increase of F-actin content (30). FPR activation has also been demonstrated to signal through the Rho family protein cell division control protein 42 (Cdc42) to activate Rac- and ARP2/3-dependent pathways leading to actin nucleation (30). Recent evidence suggests that FPR is a membrane mechanosensor that senses the mechanical fluid stress and signals intracellular cascades. Blockage of FPR reduces pseudopod retraction response to fluid stress in neutrophils (31).

It is known though, that a major mechanism by which immune system causes organ damage is due to neutrophil-mediated increases in endothelial cell permeability. Nevertheless, in a recent study from Sun and collaborators (32) demonstrated that mitochondrial DNA isolated from human liver induced dose-dependent rise in endothelial permeability both in the presence and absence of neutrophils (32). In the absence of neutrophils, however, permeability changes were transient and decayed relatively quickly. In the presence of neutrophils, the increases in permeability were sustained and even intensified over time. In addition, Sun et al. demonstrated that non-formylated mitochondrial proteins cause changes in endothelial cell permeability directly. However, bacteria-derived fMLP did not induce any change in endothelial permeability (32).

We have observed that both mitochondrial *N*-formyl peptides (formylated peptide corresponding to the NH_2_-terminus of mitochondria NADPH dehydrogenase subunit 6; F-MIT) and fMLP (bacteria derived) induce vascular leakage and exacerbated vasodilatation in resistance arteries, and that a FPR antagonist inhibits these responses (22). F-MIT, but not non-formylated peptides or mitochondrial DNA, induced severe hypotension *via* FPR activation and histamine release (22). Supporting these data, rats that underwent hemorrhagic shock increased plasma levels of F-MIT associated with lung damage, and antagonism of FPR ameliorated this response.

As described above, FPR activation leads to cytoskeleton rearrangement in immune and non-immune cells. Therefore, because FPR is present in endothelial cells and because we observed that F-MIT induces vascular leakage due to FPR activation (22), we questioned if F-MIT *via* FPR activation could lead to changes in cytoskeleton-regulating proteins in primary cultures of human aortic endothelial cells (HAEC). To answer this question, we treated HAEC with F-MIT (20 min, 10 μM) in the presence or absence of a cocktail of FPR antagonists (FPR-1 antagonist, cyclosporine H: CsH, 1 μmol/L and FPR-2 antagonist, WRW4, 10 μmol/L). Treatment with F-MIT rapidly increased RhoA/ROCK (Rho: 1.8-fold vs. Veh; ROCK: 1.4-fold vs. Veh, *p* < 0.05), CDC42 (twofold vs. Veh, *p* < 0.05), and phospho-MLC Thr/Ser19 (1.5-fold vs. Veh, *p* < 0.05) in HAEC. These changes were abolished in the presence of FPR antagonist. These data suggest that F-MIT leads to changes in endothelial cell cytoskeleton independent of leukocyte activation. Given that we provided strong evidence FPR induced RhoA and CDC42 expression, and these proteins are linked with endothelial contraction *via* actin polymerization, we infer that F-MIT elicits endothelial contraction and subsequent vascular leakage not only *via* actomyosin interaction but also *via* actin polymerization.

## Hypothesis

**Figure 1:**
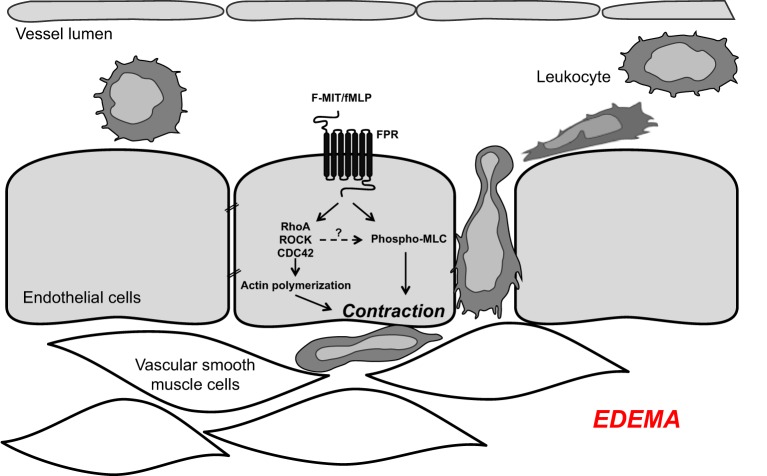
**Mitochondrial *N*-formyl peptides (FMIT) trigger endothelial cell contraction and vascular leakage *via* formyl peptide receptor (FPR) activation, contributing to the loss of control of vascular tone and edema in trauma-induced SIRS and sepsis**.

Our new mechanistic hypothesis is that F-MIT released from trauma/cell damage activate FPR leading to changes in endothelial cell cytoskeleton which subsequently induces endothelial contraction and vascular permeability (Figure 1).

### Evaluation of the Hypothesis

The inability to treat or prevent SIRS and sepsis in trauma patients may be due to our limited understanding of the molecular pathways that govern the disruption of normal vascular control and responsiveness. The proposed hypothesis is significant because it may offer a novel cellular and molecular mechanism underlying the development of arterial dysfunction in SIRS and extend our knowledge about how mitochondria-derived molecules mediate endothelial barrier dysfunction.

In the present study, we have observed that F-MIT, *via* FPR activation, elicits changes in cytoskeleton-regulating proteins in endothelial cells. Therefore, we infer that this interaction can lead to endothelial contraction, increased vascular leakage, and attenuated barrier function as observed in SIRS patients following trauma. Corroborating this hypothesis, we previously observed that F-MIT induced vascular leakage in conduit and resistance arteries from Wistar rats (22). Based on these findings, it is reasonable to speculate that in conditions of enhanced FPR activation (e.g., tissue damage/trauma), FPR-associated signaling leads increases in endothelial permeability, which subsequently contributes to end organ damage. Therefore, we believe that the FPR may be a target for the treatment of SIRS and sepsis.

## Author Contributions

CW analyzed the data in HAEC and wrote, discussed, and reviewed the manuscript. CM performed the experiments in HAEC, discussed, and reviewed the manuscript. RW reviewed the manuscript.

## Conflict of Interest Statement

The authors declare that the research was conducted in the absence of any commercial or financial relationships that could be construed as a potential conflict of interest.
